# Post-Treatment Surveillance to Detect Recurrent Disease in Esophagogastric Cancer: A Review of Current Practice and Future Perspectives

**DOI:** 10.3390/jcm15197389

**Published:** 2026-09-23

**Authors:** Nicola B. Raftery, Mark P. Ward, Claire L. Donohoe, Maeve Lowery, Jessie A. Elliott

**Affiliations:** 1 Department of Surgery, Trinity St. James Cancer Institute, St James’s Hospital, D08 NHY1 Dublin, Ireland; rafteryn@tcd.ie (N.B.R.);; 2 Department of Pharmacology and Therapeutics, Trinity College Dublin and Trinity St. James’s Cancer Institute, St James’s Hospital, D08 NHY1 Dublin, Ireland; 3 Department of Medical Oncology, Trinity St. James Cancer Institute, St James’s Hospital, D08 NHY1 Dublin, Ireland

**Keywords:** esophagogastric adenocarcinoma, postoperative surveillance, survivorship, imaging, cancer recurrence

## Abstract

Effective post-treatment surveillance in cancer is designed to drive early detection and treatment of recurrence; however, surveillance protocols following curative-intent treatment for esophagogastric adenocarcinoma (EGA) remain poorly defined, with no consensus across existing guidelines. Historically, a lack of evidence regarding optimal surveillance approaches has been underpinned by limited treatment efficacy in recurrent or metastatic disease. However, recent advances in chemotherapy, immunotherapy, and targeted approaches, as well as the management of oligometastatic disease, have transformed the therapeutic landscape, making early detection of recurrence increasingly actionable. The rising incidence of EGA in younger patients adds further urgency to understanding long-term surveillance and survivorship needs. While ongoing clinical trials seek to address this question, emerging technologies, including liquid biopsy, next-generation sequencing (NGS), and artificial intelligence (AI), promise to further advance surveillance strategies. This review explores the rationale for post-treatment surveillance in EGA and examines future technologies that will shape surveillance strategies into the future.

## 1. Introduction

Globally, gastric and esophageal adenocarcinoma (from here on referred to as esophagogastric adenocarcinoma (EGA)) represents one of the most common and aggressive cancer types, with approximately 1.6 million new cases and over 700,000 deaths reported annually [1,2,3].

Recent advances in the multimodal management of EGA have led to major improvements in outcomes, with 5-year overall survival (OS) approximately doubling from historical estimates of ~10–15% to approximately ~20–25% in contemporary series [1,2]. Despite this progress, challenges remain, as many patients present with advanced-stage disease, with over half diagnosed with metastatic involvement that limits curative treatment options. Furthermore, even among patients treated with curative intent, recurrence occurs in up to 50% in modern series, most within three years [3].

However, emerging therapies, including next-generation HER2-targeting agents, immunotherapy, claudin 18.2 directed therapies, FGFR2b targeting agents, stereotactic radiotherapy, local ablative approaches, and surgery for oligometastatic disease, are beginning to expand treatment possibilities in the recurrent and metastatic setting. The availability of effective therapies for the management of recurrent and metastatic disease increases the rationale for intensive post-treatment surveillance; however, limited studies to date have assessed the impact of surveillance strategies on outcomes following cancer recurrence.

In this review, we explore current evidence regarding surveillance strategies in EGA and consider future directions in surveillance and survivorship, incorporating insights from ongoing clinical trials and emerging technologies.

### Literature Search and Review Methodology

This narrative review was informed primarily by PubMed/MEDLINE searches conducted from November 2025 to September 2026. Search terms included combinations of “esophageal cancer”, “gastric cancer”, “gastroesophageal junction cancer”, “surveillance”, “follow-up”, “recurrence”, “imaging”, “endoscopy”, “ctDNA”, “liquid biopsy”, “next-generation sequencing”, “artificial intelligence”, and related terms. Targeted searches were also undertaken for international clinical guidelines and relevant clinical trials with a focus on surveillance following curative-intent treatment. Studies evaluating surveillance after endoscopic therapy or definitive chemoradiotherapy, as well as those evaluating on-treatment monitoring in advanced and metastatic disease, were considered beyond the scope of this article.

Publications were selected based on relevance to post-treatment surveillance following curative-intent treatment, with emphasis on clinical guidelines, systematic reviews, prospective studies, large observational studies, and emerging surveillance technologies.

## 2. Changing Therapeutic Landscape: Rationale for Surveillance

Historically, surveillance in EGA was considered of limited benefit, as salvage therapies following recurrence, particularly distant metastasis, offered little advantage. This nihilistic perspective fostered a conservative approach to follow-up in many health systems, an approach that is reflected in current clinical guidelines, particularly in Europe [4,5,6]. However, for patients undergoing treatment with curative intent, over 90% of recurrences occur within 36 months, with about 50% within the first year, highlighting a critical window for intervention [3,7]. Advances in systemic therapies and molecular oncology are shifting this paradigm, expanding the potential for effective treatment of recurrent disease.

The therapeutic landscape for recurrent or metastatic EGA has evolved significantly in recent years. Immunotherapy has demonstrated a clear survival benefit in subsets of patients, particularly those with PD-L1 positivity or microsatellite instability, while actionable biomarkers such as HER2 and Claudin 18.2 can guide targeted therapies. These developments have transformed the management of recurrent and metastatic disease, particularly among patients with a good performance status. As a result, there is now an emerging rationale for active surveillance following curative-intent treatment. With the emergence of several biomarker-directed therapeutic approaches, future surveillance protocols may be tailored according to biomarker status and should include strategies for biomarker profiling to guide further therapy. Future studies may assess whether more intensive surveillance strategies are of differential benefit according to biomarker status of the primary tumor.

### 2.1. HER2-Directed Therapies

The first targeted therapy approved for use in EGA was trastuzumab, representing a paradigm shift in the management of advanced and metastatic disease in this setting. HER2 (human epidermal growth factor receptor 2) is a transmembrane tyrosine kinase receptor encoded by the ERBB2 gene. Overexpression or gene amplification of HER2 occurs in approximately 20% of EGA. Its clinical significance in EGA was established by the landmark ToGA trial, which demonstrated that adding trastuzumab to chemotherapy in HER2-positive advanced gastric/GEJ cancers improved overall survival (13.8 vs. 11.1 months), leading to trastuzumab’s incorporation as a first-line standard of care [8]. Following this, trials such as DESTINY-Gastric01 and KEYNOTE-811 have established roles for the antibody–drug conjugate trastuzumab deruxtecan (T-DXd) in later line therapy and for trastuzumab plus pembrolizumab in the first-line setting for patients with PD-L1-positive disease [9,10]. From a surveillance standpoint, HER2-positive tumors, while historically considered more aggressive, are now therapeutically actionable, which shifts the focus of surveillance toward detecting recurrence early enough for targeted reintervention. Future directions include continued development of HER2 antibody–drug conjugates, bispecific antibodies, investigation of targeted therapies for HER2-low populations, and liquid-biopsy-based HER2 evaluation.

### 2.2. Immunotherapy

PD-L1 (Programmed Death-Ligand 1) is a transmembrane protein expressed on tumor cells, antigen-presenting cells, and other cells within the tumor microenvironment. It binds PD-1 (Programmed Death-1) receptors on activated T cells, delivering an inhibitory signal that suppresses T-cell proliferation, cytokine production, and cytotoxic activity.

Tumors exploit this pathway as a mechanism of immune evasion, upregulating PD-L1 to inactivate tumor-infiltrating lymphocytes. This is the biological rationale underpinning PD-1/PD-L1 checkpoint inhibitors, which block this interaction and restore anti-tumor T-cell activity. In EGA, PD-L1 expression may be assessed using scoring systems including the Combined Positive Score (CPS), Tumor Proportion Score (TPS), and Tumor Area Positivity (TAP), depending on tumor type, therapeutic agent, and assay. CPS ≥ 1 is found in a majority of advanced gastric and junctional tumors, with higher CPS correlating to a greater likelihood of response to ICIs [11]. The landmark KEYNOTE-590, -061, -062, -859, and CheckMate-649 trials demonstrated greater benefit from anti-PD-1 therapies such as pembrolizumab and nivolumab with increasing PD-L1 expression, although approved indications and PD-L1 thresholds vary according to therapeutic agent and jurisdiction [12,13,14,15,16]. Furthermore, following the publication of the landmark MATTERHORN trial, combination chemoimmunotherapy with durvalumab and FLOT has become the standard neoadjuvant approach for patients with locally advanced tumors, thus moving immunotherapy biomarker testing to the preoperative setting [17]. This has potential implications for post-treatment surveillance, as PD-L1 status may influence anticipatory planning for use of immunotherapy in case of recurrence, while little is known about the evolution of immunotherapy resistance following recurrence among pretreated patients.

In addition, microsatellite instability (MSI) is a molecular phenotype arising from deficiency in the DNA mismatch repair (MMR) system, leading to the accumulation of insertion/deletion type errors in microsatellite regions of the DNA. Tumors may be classified as microsatellite instability-high (MSI-H), microsatellite instability-low (MSI-L), or microsatellite stable (MSS). The Cancer Genome Atlas (TCGA) has identified MSI-H as one of the four major molecular subtypes of esophagogastric cancer [18]. MSI-H is highly predictive of response to immunotherapy, and MSI-H represents the first FDA-approved tumor-agnostic biomarker for immunotherapy following the KEYNOTE-158, KEYNOTE-164, and KEYNOTE-051 trials [19,20,21]. MSI-H status has been shown to be associated with durable responses to immune checkpoint inhibitors (ICIs), as well as with resistance to 5-fluorouracil-based chemotherapy [22]. In line with this, the phase II INFINITY trial demonstrated that neoadjuvant tremelimumab plus durvalumab achieved high pathological and clinical response rates in resectable MSI-H EGA, suggesting that chemotherapy-free approaches and even non-operative management may be feasible for select patients [23]. As such, MSI status should be tested upfront to guide therapy in EGA. As we look toward future surveillance strategies, taking into consideration the advancing and increasingly standardized molecular classification of tumors, surveillance may in the future become risk-adapted and biologically informed. Prospective studies on surveillance intensity stratified according to molecular status may be informative.

### 2.3. FGFR2b-Directed Therapy

FGFR2b (fibroblast growth factor receptor 2b) is a splice variant of the FGFR2 gene, with recent reports of overexpression in 37.8% of patients with gastric cancer [24,25]. It plays a role in tumorigenesis via activation of the FGFR pathway, leading to cell proliferation, angiogenesis, and survival [18]. The phase II FIGHT trial brought FGFR2b to the forefront by demonstrating that bemarituzumab, a monoclonal antibody targeting FGFR2b, when added to standard chemotherapy, significantly prolonged progression-free survival (PFS) and overall survival (OS) in patients with FGFR2b-overexpressing advanced gastric/junctional cancers [19,20]. These findings prompted phase III trial evaluation with combination of bemarituzumab with standard chemotherapy and standard chemotherapy plus immunotherapy (FORTITUDE-101 and FOTRITUDE-102, NCT05052801 and NCT05111626). In FORTITUDE-101, bemarituzumab plus mFOLFOX6 met the primary OS endpoint in patients with FGFR2b overexpression (2+/3+ staining in ≥10% of tumor cells), with a median OS of 17.9 versus 12.5 months with placebo plus mFOLFOX6 (HR 0.61, 95% CI 0.43–0.86). However, this benefit attenuated with longer follow-up, with median OS of 14.5 versus 13.2 months (HR 0.82, 95% CI 0.62–1.08) in a subsequent descriptive analysis [21]. Furthermore, FORTITUDE-102 (NCT05111626), which evaluated bemarituzumab in combination with chemotherapy and nivolumab, was stopped early due to inadequate efficacy [22]. Consequently, the role of FGFR2b-directed therapy in OGA cancer remains uncertain pending further analysis of the available data and future trials.

### 2.4. Claudin-Directed Therapy

Claudin 18.2 is a tight junction protein normally restricted to gastric mucosa but aberrantly exposed on tumor cell membranes in approximately ~38% of gastric cancers [23]. The recent development of zolbetuximab, a monoclonal antibody targeting CLDN18.2, has led to much excitement about the potential of Claudin18.2 as an actionable biomarker in gastric and junctional cancers. The SPOTLIGHT trial showed that adding zolbetuximab to FOLFOX in CLDN18.2-positive, HER2-negative advanced gastric/junctional cancer improved the median OS from 15.5 to 18.2 months [26]. The GLOW trial also demonstrated improved PFS (6.8 vs. 8.2 months) and OS (12.16 vs. 14.39 months) when zolbetuximab was added to CAPOX chemotherapy [27]. These trials have led to zolbetuximab being considered first-line standard of care for CLDN18.2-positive, HER2-negative gastric/junctional adenocarcinoma, with both European Medicines Agency (EMA) and Food and Drug Administration (FDA) approval in 2024 and subsequent inclusion in clinical guidelines [28,29].

### 2.5. Treatment of Oligometastatic Disease and Surveillance

An important evolution in the management of EGA has been the recognition of oligometastatic disease (OMD) as a potentially treatable stage, with this international mindset shift reflected in the publication of the OMEC guidelines [30]. Advances in systemic and local therapy techniques may now allow for the aggressive treatment of limited metastatic disease with curative or life-prolonging intent. These interventions are contingent on early and accurate detection of recurrence before the disease process becomes polymetastatic. The shift toward treating oligometastatic disease reinforces the rationale for structured surveillance strategies integrated into the broader continuum of care. However, at present, no level 1 evidence exists to support an intensive surveillance approach in EGA.

## 3. Components of an Effective Surveillance Strategy

When considering what would constitute a successful surveillance strategy in EGA, one must weigh the intended goals of surveillance against the tools available while accounting for cost-effectiveness and acceptability to patients. Ideally, surveillance would detect recurrence early enough to permit effective intervention, preserve performance status, and maintain or improve health-related quality of life (HRQoL). Current components of a surveillance strategy may include clinical review, symptom and patient-reported outcome monitoring, cross-sectional imaging, endoscopy, blood-based tumor markers, liquid biopsy, and nutritional assessment.

What does success look like? Two questions underpin this: what are the goals of surveillance, and what tools are currently available to meet them? Goals to consider include whether surveillance itself improves HRQoL or provides reassurance, whether effective treatment exists for detected recurrence (in terms of tumor characteristics, patient performance status, and patient preference), and whether earlier detection may preserve performance status, ultimately improve overall survival, or increase the likelihood of cure. On the other hand, a surveillance strategy that identifies recurrence earlier without altering the treatment options available, or without a demonstrable survival benefit, may negatively impact HRQoL and increase anxiety [31].

Surveillance and earlier detection of recurrence, by definition, lengthens the interval between diagnosis and death, even if the natural history and ultimate outcome of the disease are unchanged (Figure 1). Apparent improvements in survival from intensive surveillance can therefore be an artifact of earlier labelling rather than a true therapeutic benefit—a phenomenon known as lead time bias, and any evaluation of surveillance strategy must be designed and interpreted with this bias explicitly in mind, ideally through randomized comparison of surveillance intensity with survival and HRQoL as co-primary endpoints rather than time-to-recurrence detection alone [32].

Interpretation of observational surveillance studies may be further complicated by other forms of bias, including length–time bias, selection bias, and confounding by indication [33]. For example, surveillance may preferentially detect more indolent recurrences, while patients undergoing intensive follow-up may differ systematically in disease characteristics, performance status, and eligibility for salvage treatment. These factors may create an apparent association between surveillance intensity and improved outcomes independent of any true surveillance effect. Consequently, earlier recurrence detection or longer post-recurrence survival cannot establish clinical benefit; appropriately controlled prospective studies evaluating overall survival are required. Furthermore, for a surveillance modality to be clinically effective, earlier detection of recurrence must translate into a meaningful change in management and improved patient outcomes.

Equally, the goals of surveillance cannot be considered in isolation from its burden on patients and caregivers. Intensive surveillance carries a recognized psychological cost, with scan-associated anxiety a well-described phenomenon in oncological follow-up, and the cumulative burden of repeated appointments, imaging, and blood tests may itself detract from HRQoL regardless of oncological outcome, reinforcing a persistent illness identity and contributing to fear of cancer recurrence. There is also an economic dimension, both to the patient and to the health system, which must be weighed against the clinical benefit of any given surveillance component. An effective strategy is therefore one that allows preservation of performance status, creates a meaningful clinical benefit at the time of recurrence detection, and positively impacts HRQoL and survival.

Candidate components include CT and PET scanning, endoscopy, blood-based tumor markers, and liquid biopsy. Nutritional assessment merits inclusion, with performance status and HRQoL both identified above as central goals of surveillance. Both are directly influenced by nutritional status in this patient group, and structured nutritional review offers a low-cost means of addressing a goal of surveillance that is independent of that achieved through oncologic follow-up alone [34].

## 4. Current International Surveillance Practices in EGA

Despite significant progress in the multidisciplinary management of EGA, with increasing international consensus regarding optimal perioperative and surgical strategies, approaches to surveillance following treatment with curative intent remain inconsistent. Current guidelines offer varied recommendations regarding the utility, frequency, modality, and duration of post-treatment surveillance in this context (Table 1). This heterogeneity largely reflects heretofore a lack of evidence regarding the impact of surveillance strategies on long-term oncologic outcomes and HRQoL, in addition to differences in healthcare systems and reimbursement models, cultural expectations regarding cancer survivorship care, and varied international perspectives regarding the potential benefit of salvage therapies in the context of recurrent or metastatic disease. In contrast to other cancer types, where surveillance protocols are supported by level 1 evidence, post-treatment monitoring in EGA often depends on local guidelines and institutional and clinician preferences, highlighting a critical gap in evidence-based consensus [32,35,36].

Current guideline recommendations vary according to primary tumor site, disease stage, and treatment modality, reflecting differences in recurrence patterns and the availability of local salvage strategies. These distinctions are therefore retained in the comparison below rather than presenting surveillance recommendations as a single uniform EGA pathway (Table 1).

Current international guidelines demonstrate substantial variation in post-treatment surveillance recommendations for esophageal, gastroesophageal junction (GEJ), and gastric cancer (Table 1), reflecting both differences in disease site and treatment modality and the limited evidence supporting a single optimal surveillance strategy. European Society for Medical Oncology (ESMO) guidelines for esophageal cancer note that routine follow-up has not been shown to improve survival in patients without the potential for endoscopic reintervention or salvage treatment and recommend that follow-up focus on symptoms, nutritional status, and psychosocial support [V, A]. No fixed postoperative clinical, radiological, or endoscopic surveillance schedule is specified [4]. Similarly, ESMO gastric cancer guidelines recommend regular follow-up for investigation and treatment of symptoms, psychological support, and early detection of recurrence [III, B], with follow-up individualized according to the patient and disease stage [V, B], but do not prescribe a fixed post-curative imaging or endoscopic surveillance schedule [5].

The National Comprehensive Cancer Network (NCCN) provides more prescriptive stage- and treatment-specific recommendations. For esophageal and esophagogastric junction cancer, NCCN acknowledges that surveillance following successful treatment remains controversial and that recommendations are based largely on retrospective evidence and expert consensus [37]. Surveillance differs substantially according to whether patients have undergone endoscopic therapy or esophagectomy. Following endoscopic treatment of superficial disease, surveillance is predominantly endoscopic, whereas CT surveillance is incorporated for higher-risk disease following esophagectomy. NCCN gastric cancer recommendations are similarly stage- and treatment-specific. Following treatment of Tis or pStage I disease, CT is generally performed according to clinical indication, whereas patients with pStage II–III or ypStage I–III disease following neoadjuvant ± adjuvant therapy undergo CT of the chest, abdomen, and pelvis every 6 months for the first 2 years and annually thereafter up to 5 years [38]. Endoscopic surveillance also varies according to whether treatment consisted of partial or subtotal gastrectomy or total gastrectomy.

A different approach is evident in Japanese guidelines. The Japanese Gastric Cancer Association (JGCA) provides structured stage-specific postoperative surveillance schedules, with clinical assessment and scheduled imaging for Stage II–III. Nevertheless, the guideline acknowledges that there is little evidence that systematic postoperative surveillance prolongs survival and that prospective evidence defining the optimal surveillance modalities and intervals is lacking [39]. The Japan Esophageal Society (JES) similarly considers regular follow-up important following radical resection but does not establish an evidence-based standard postoperative surveillance schedule. The applicability of JES recommendations to EAC should be interpreted cautiously because the underlying Japanese evidence base predominantly reflects ESCC [40,41].

**Table 1:** Summary of selected international guideline recommendations for post-treatment surveillance following curative-intent treatment of esophageal, gastroesophageal junction, and gastric cancer.

Guideline/Society	Version/Year	Disease Site/Treatment Population	Follow-Up Visits; History, Physical Exam	Imaging	Endoscopy	Labs/Biomarkers	Duration of Surveillance	Notes
NCCN	Esophageal and Esophagogastric Junction Cancers, v3.2026 [37]	Esophageal and EGJ cancer following successful local therapy; surveillance stratified by tumor stage and treatment modality.	No fixed routine clinical-visit schedule specified; follow-up is individualised according to tumor stage and treatment modality. Routine medical care and survivorship assessment should continue as clinically appropriate.	Tis/T1a after Endoscopic Resection (ER)/ablation or oesophagectomy: routine surveillance imaging not recommended. T1b N0 after ER/ablation: CT chest/abdomen may be considered every 6 months for 2 years, then annually up to 5 years. T1b or greater/any N or T1a N+ after oesophagectomy ± adjuvant therapy: CT chest/abdomen should be considered every 6 months for 2 years, then annually up to 5 years. Any T/any N after neoadjuvant therapy or chemoradiotherapy followed by oesophagectomy (±adjuvant treatment): CT chest/abdomen should be considered every 6 months for up to 2 years, then annually up to 5 years.	Tis/T1a after ER/ablation: EGD every 3 months in year 1, every 6 months in year 2, then annually indefinitely. T1b N0 after ER/ablation: EGD every 3 months in year 1, every 4–6 months in year 2, then annually indefinitely; EUS may be considered. After oesophagectomy ± adjuvant therapy: EGD as needed based on symptoms/radiographic findings; incompletely resected Barrett’s oesophagus requires ablation and scheduled surveillance. After neoadjuvant therapy or chemoradiotherapy followed by oesophagectomy: EGD as clinically indicated.	No routine tumor-marker surveillance schedule specified	Cancer-specific surveillance generally up to 5 years; endoscopic surveillance after ER/ablation of superficial disease continues indefinitely.	NCCN states that surveillance remains controversial, with no high-level evidence defining the optimal algorithm; recommendations are stage- and treatment-specific and based on retrospective evidence and panel expertise. All recommendations are Category 2A unless otherwise indicated.
NCCN	Gastric Cancer, v3.2026 [38]	Gastric cancer following curative-intent treatment; surveillance stratified by pathological stage and treatment modality.	Clinical examination every 3–6 months for 1–2 years, then every 6–12 months for years 3–5.	Tis after ER: CT chest/abdomen/pelvis as clinically indicated based on symptoms or concern for recurrence. pStage I: CT chest/abdomen/pelvis as clinically indicated. pStage II/III or ypStage I–III after neoadjuvant ± adjuvant therapy: CT chest/abdomen/pelvis every 6 months for the first 2 years, then annually for up to 5 years.	Tis after ER: EGD every 6 months for 1 year, then annually for 3 years. pStage I treated by ER: EGD every 6 months for 1 year, then annually for up to 5 years; thereafter as needed according to symptoms/radiographic findings. pStage I after surgical resection: EGD as clinically indicated. pStage II/III or ypStage I–III after partial/subtotal gastrectomy: EGD as clinically indicated. Following total gastrectomy endoscopy is performed only as clinically indicated in symptomatic patients.	Monitor for and treat nutritional deficiencies following surgical resection, particularly after total gastrectomy.	Routine gastric cancer surveillance through 5 years; additional follow-up after 5 years may be considered according to risk factors and comorbidities. Lifelong monitoring and management of nutritional sequelae following gastrectomy.	Surveillance intensity is stage- and treatment-specific. CT is preferred for scheduled imaging; PET/CT or MRI may be used when CT cannot be performed, as clinically indicated. All recommendations are Category 2A unless otherwise indicated.
ESMO	2022 Clinical Practice Guideline [5]	Gastric cancer following curative-intent treatment	Regular follow-up is recommended for investigation and treatment of symptoms, psychological support and early detection of recurrence [III, B]. Follow-up should be individualised according to the patient and disease stage [V, B]. Nutritional support, including assessment and management of vitamin and mineral deficiencies, is recommended [V, B].	No fixed routine post-curative imaging schedule specified.	Endoscopy & biopsies as clinically indicated	No routine tumor-marker surveillance schedule specified	No fixed duration	No prescriptive post-curative surveillance schedule is provided.
ESMO	2022 Clinical Practice Guideline [4]	Esophageal cancer following curative-intent therapy	Follow-up should focus on symptoms, nutritional status and psychosocial support [V, A]. No fixed routine visit schedule is specified.	No routine postoperative imaging interval is specified.	No routine postoperative endoscopic interval is specified.	No routine serum biomarker surveillance schedule specified.	No fixed surveillance duration specified.	Guideline notes that routine follow-up has not been shown to improve survival.
Japanese Esophageal Society (JES)	Esophageal cancer practice guidelines 2022 edited by the Japan Esophageal Society: part 2. Guideline (published 2023) [40]	Esophageal cancer following radical resection	Post-resection: regular follow-up is considered important, but no standard schedule is established.	Post-resection: no evidence-based imaging interval established.	Post-resection: regular upper GI endoscopy is advised for surveillance of the pharynx, remnant oesophagus and stomach/gastric tube, including detection of metachronous malignancy; no evidence-based interval established.	No routine tumor-marker surveillance schedule specified	No standard evidence-based duration after radical resection; institutional surveillance may extend beyond 5 years.	No high-level evidence establishes the survival benefit or optimal surveillance method following radical resection.
Japanese Gastric Cancer Association (JGCA), 2025, 7th Edition	Japanese Gastric Cancer Treatment Guidelines 2025, 7th edition (English version published 2026) [39]	Gastric cancer following curative-intent treatment; surveillance stratified by pathological stage (Stage I vs. Stage II–III) and by use of endoscopic resection	Stage I: clinical examination at 1, 6 and 12 months, then every 6 months to 3 years and annually at years 4–5. Stage II–III: every 3 months for 2 years, then every 6 months to 5 years.	Stage I: CT and/or US as necessary. Stage II–III: CT and/or US at 6, 12, 18 and 24 months, then at 2.5, 3, 4 and 5 years. Additional imaging (including PET) as clinically indicated.	Examination of the remnant stomach as necessary.	Blood tests including tumor markers follow the same stage-specific schedule as clinical assessment. The guideline identifies CEA and CA19-9 as useful tumor markers;	5 years according to the reference schedules; individualised surveillance thereafter.	Guideline acknowledges that there is little evidence that systematic postoperative surveillance prolongs survival, and no prospective evidence establishes the optimal surveillance modalities or intervals.
NICE	NG83 (2018; last updated 2023) [6]	Esophagogastric cancer following curative-intent treatment	Do not offer routine clinical follow-up solely to detect recurrent disease. Provide information about symptoms of recurrence and offer rapid access to the esophago-gastric MDT for review if symptoms develop.	No routine radiological surveillance schedule specified.	No routine endoscopic surveillance schedule specified.	No routine tumor-marker surveillance schedule specified	No fixed schedule	Symptom-triggered rather than routine recurrence surveillance. Recommendations apply specifically to asymptomatic patients without evidence of residual disease after curative-intent treatment.

CA19-9, carbohydrate antigen 19-9; CEA, carcinoembryonic antigen; CR, complete response; CRT, chemoradiotherapy; CT, computed tomography; EAC, esophageal adenocarcinoma; EGD, esophagogastroduodenoscopy; EGJ, esophagogastric junction; ER, endoscopic resection; ESCC, esophageal squamous cell carcinoma; ESMO, European Society for Medical Oncology; EUS, endoscopic ultrasound; JGCA, Japanese Gastric Cancer Association; JES, Japan Esophageal Society; MDT, multidisciplinary team; NCCN, National Comprehensive Cancer Network; NICE, National Institute for Health and Care Excellence; PET/CT, positron emission tomography/computed tomography; US, ultrasonography. Evidence grading: Where reported, evidence levels and recommendation grades are reproduced according to the grading system used by the respective guideline; grading systems are not directly comparable between organizations.

The United Kingdom National Institute for Health and Care Excellence (NICE) adopts a less intensive, predominantly symptom-triggered approach. For asymptomatic patients without evidence of residual disease following curative-intent treatment, NICE recommends providing information regarding symptoms of recurrence and rapid access to the esophagogastric multidisciplinary team if symptoms develop while specifically advising against routine clinical or radiological follow-up solely for the detection of recurrent disease [6].

Collectively, these differences demonstrate the absence of international consensus regarding the optimal modality, frequency, and duration of post-treatment surveillance. Recommendations remain stratified by primary tumor site and disease stage despite the absence of clear evidence for this approach, with the largest previous study in this area, further discussed below, showing that patients with favorable histopathologic features and those treated with upfront surgery rather than neoadjuvant therapy exhibited the greatest benefit from intensive surveillance. These data, though non-randomized, suggest that tumor biology and sensitivity to systemic therapies may be a key predictor of benefit from intensified surveillance, and data from ongoing randomized trials in this area are eagerly awaited to inform guideline development and understand whether stratified surveillance is appropriate and for whom. Several guidelines explicitly acknowledge the limited high-level evidence demonstrating that intensive surveillance improves survival (Table 1).

Previous data from the European Registration of Cancer Care (EURECCA) Upper Gastrointestinal group demonstrate substantial variation in postoperative surveillance practices in EGA across European centers. In a survey of 10 centers, routine cross-sectional imaging was performed in approximately 40% of patients and tumor markers were used in about 40%, while details regarding endoscopic surveillance and duration of follow-up were not reported [42]. Subsequent feasibility data from the ENSURE collaborative survey of 18 European centers showed similar heterogeneity, with 39% of centers performing routine cross-sectional imaging and 11% utilizing PET–CT for surveillance, with follow-up decisions more commonly driven by clinical indicators such as weight loss or symptoms rather than pathological stage, response to neoadjuvant therapy, or published guidelines [31]. A similar distinction between the limited evidence base and real-world surveillance practice is evident in Japan. The 2022 Japan Esophageal Society (JES) guideline reports findings from a 2020 nationwide survey of JES-accredited institutions evaluating follow-up after radical oesophagectomy [40,43]. Among patients with pStage II–IV disease, medical interview and physical examination were performed at least five times annually during the first postoperative year in 64% of institutions, while cervical-to-pelvic CT was performed at least three times annually in 61%. By postoperative year 5, CT continued to be performed at least annually in 96% of institutions. Upper gastrointestinal endoscopy was performed at least annually in almost all institutions through postoperative year 5, with surveillance frequently continuing beyond five years. Despite the intensity of surveillance reported in this nationwide survey, the JES guideline does not specify a standard postoperative surveillance timeline following radical resection, reflecting the absence of high-level evidence defining an optimal follow-up strategy or demonstrating a survival benefit [40]. These data should be interpreted in the context of the Japanese esophageal cancer population, in which ESCC predominates, limiting direct extrapolation to EAC [41].

## 5. Current Evidence for Post-Treatment Surveillance in EGA

Disparities between current international guidelines regarding post-treatment surveillance likely reflect historically limited high-quality evidence to address this question. Studies to date have been limited by their retrospective and observational nature, with no published randomized controlled trials available to inform clinical decision-making. Furthermore, the surveillance literature is uniquely impacted by reporting bias, in particular arising from lead time bias with respect to recurrence detection, and post-recurrence survival (Figure 2).

A recent systematic review and meta-analysis highlights the pitfalls of lead time and reporting bias in the interpretation of surveillance outcomes in EGA, illustrating important limitations that complicate the development of evidence-based surveillance guidelines [3]. In this report, which included 15 studies comprising some 6271 patients, the authors noted that imaging surveillance was associated with earlier detection of recurrence in esophageal cancer, as well as improved post-recurrence survival in both esophageal and gastric cancer by approximately 14 months (95% CI 1.40–27.26; *p* = 0.03). However, remarkably, it was not possible based on the current reported literature to determine whether post-treatment surveillance was associated with improvements in overall survival time or increased probability of long-term cure due to variability in outcome reporting [3]. The lack of reporting of overall survival time and survival probabilities within the published literature highlights the risks associated with reporting bias—intensive imaging surveillance was associated with increased recurrence detection and increased post-recurrence survival, without a clear overall survival benefit, suggesting that radiologic follow-up may simply reduce the lead time to diagnosis of recurrence, without objectively impacting disease trajectory or long-term outcomes. Careful examination of the surveillance literature is essential to limit the risk that reporting bias impacts interpretation of study findings.

The largest study in this area to date specifically addressing postoperative surveillance following esophageal and junctional cancer is the ENSURE study, published in 2023. This international multicenter cohort study included 4682 patients across 20 European and North American centers [31]. Of the centers included in the study, 37% adopted an intensive surveillance approach, which was defined as the use of cross-sectional surveillance imaging at least annually for 3 postoperative years, while 18.9% utilized surveillance endoscopy. The primary endpoint of the study was overall survival (OS), and the median follow-up was 60 months, with a 48% recurrence rate across the entire study population, highlighting the maturity of the outcome data. ENSURE demonstrated that, among all patients, although intensive surveillance follow-up was associated with increased receipt of tumor-directed therapy for recurrence among patients with EGA, this did not result in increased overall survival across the entire study cohort (HR 1.01 [95% confidence interval 0.89–1.13]). Subgroup analysis suggested that patients treated with upfront surgery and those with (y)pTis-2 disease may benefit from imaging surveillance, with improved overall survival following intensive follow-up in these cohorts, perhaps indicating a greater likelihood of successful salvage therapies for recurrence in this context [34].

Importantly, intensive imaging surveillance was not associated with any overall difference in health-related quality of life (HR-QL); however, imaging surveillance was associated with increased health anxiety scores, suggesting a potential drawback of intensive surveillance strategies in reinforcing a persistent illness identity or increasing fear of cancer recurrence. In contrast, intensive surveillance was associated with improved scores across some aspects of eating symptoms, perhaps indicating a downstream improvement in the management of functional disorders with a more structured follow-up approach.

Surveillance endoscopy is similarly inconsistent in international application, reflecting the same paucity of high-quality evidence that limits imaging-based protocols. Practice varies significantly between healthcare systems, with endoscopic surveillance undertaken in over 80% of patients in Japan, compared with approximately 6% to 19% of patients in most European and North American series. In Eastern series, this may be driven by an increased prevalence of generalized field change with residual pre-neoplasia common following both surgery for esophageal squamous cell carcinoma and function-preserving gastrectomy for early gastric cancer, in addition to a well-established culture of endoscopic screening and surveillance. In the European context, ENSURE showed that just 18.9% of centers adopted routine post-treatment endoscopy, most often in conjunction with cross-sectional imaging rather than as a standalone strategy [31].

In ENSURE, endoscopic surveillance was independently associated with a reduced rate of symptomatic recurrence and an increased probability of isolated local or anastomotic recurrence, likely reflecting the sensitivity of endoscopy for subclinical disease at the anastomosis. Importantly, however, endoscopic surveillance conferred no overall or disease-specific survival benefit across the study population as a whole, and isolated local or anastomotic recurrence detected only by endoscopy was uncommon. A benefit was, however, observed in the subgroup of patients with a prior diagnosis of Barrett’s oesophagus, among whom endoscopic surveillance was independently associated with improved overall survival. It remains to be determined whether this finding was driven by patients with residual Barrett’s esophagus at the margin following resection, in whom a clear incentive for endoscopic surveillance exists, or the wider cohort of patients with a history of Barrett’s, indicating the potential for further intestinal metaplasia-associated carcinogenesis in the esophageal remnant. Nevertheless, this finding lends support to a risk-stratified approach, in which endoscopic surveillance is reserved for patients with recognized risk factors for an isolated local or anastomotic recurrence, reinforcing the broader principle that surveillance intensity should be matched to disease biology. Although subgroup analyses from ENSURE suggested that patients undergoing upfront surgery and those with favorable pathological features may derive greater benefit from intensive surveillance, these findings are non-randomized and hypothesis-generating [31]. Prospective evidence from the ongoing SARONG trials is awaited before a risk-stratified surveillance approach can be recommended. Future randomized controlled trials should seek to clarify the role of surveillance endoscopy following surgical resection, and in particular which subgroups may derive benefit from this approach.

## 6. Surveillance After Resection of Oesophageal and Gastric Cancer (SARONG) I and II Trials

The Surveillance After Resection of Oesophageal and Gastric Cancer (SARONG) I (ISRCTN14417629) and II (NCT06115629) international multicenter randomized controlled trials seek to address whether intensive imaging and endoscopic surveillance improves outcomes as compared with symptom-driven surveillance in following curative-intent surgical treatment for EGA (Figure 2) [44].

These paired UK and EU trials are open-label, multicenter, two-arm parallel randomized controlled trials evaluating intensive postoperative surveillance versus standard follow-up after curative-intent resection for EGA. Eligible patients are randomized 1:1 postoperatively into the imaging surveillance arm or the symptom surveillance arm. The imaging surveillance arm undergoes clinical review plus CT-based imaging every 6 months for 3 years with endoscopy at 12 months, whereas the symptom surveillance arm receives usual postoperative clinical follow-up every 6 months for 3 years, with investigations triggered by symptoms or clinical concern. The primary outcome is 3-year all-cause mortality, while key secondary outcomes include disease-specific mortality, patterns and treatment of recurrence, HRQL including anxiety, and cost-effectiveness, with a target sample size of 952 patients in each trial and planned follow-up to 36 months after randomization. Once completed, the SARONG trials will represent the first available randomized-trial-level evidence for surveillance in EGA and will be crucial for future clinical guideline updates. SARONG-I UK has recently completed recruitment, while the EU SARONG-II trial continues to recruit and is currently open in Ireland, Sweden, Norway, Germany, and Italy.

## 7. Future Directions in Post-Treatment Surveillance

While the SARONG trials will determine the role of imaging surveillance as compared with symptomatic follow-up in EGA, emerging technologies such as circulating tumor DNA (ctDNA) and other blood-based biomarkers offer the potential for earlier and less invasive detection of recurrence, possibly identifying minimal residual disease (MRD) before it becomes radiologically apparent. These liquid biopsy approaches represent a rapidly emerging area of investigation (Figure 3).

### 7.1. ctDNA in EGA

ctDNA as a method of liquid biopsy has emerged as a promising prognostic and predictive tool in cancer management. ctDNA comprises fragments of DNA shed by tumor cells into the bloodstream that can be detected using highly sensitive molecular techniques such as digital droplet polymerase chain reaction (ddPCR) and next-generation sequencing (NGS). ctDNA has been shown to have predictive value for recurrence in colon cancer, and following large clinical trials, it is now increasingly part of adjuvant treatment risk stratification in postoperative patients with stage II and III disease [7,8,45]. Ococks et al. demonstrated that ctDNA was predictive of relapse among patients with esophageal adenocarcinoma and also showed that median cancer-specific survival was significantly less for patients with detectable ctDNA during follow-up (10.0 months vs. 29.9 months, respectively, hazard ratio 5.55, 95% confidence interval 2.42–12.71; *p* = 0.0003) [46]. In the PLAGAST biomarker study, longitudinal ctDNA monitoring in patients with locally advanced resectable gastric and junctional adenocarcinoma demonstrated strong prognostic value with ctDNA positivity at any timepoint associated with significantly worse recurrence-free survival (RFS) and OS, with the highest risk seen in patients who remained ctDNA-positive throughout treatment (RFS HR 18.57). Early ctDNA clearance predicted better outcomes, highlighting the potential for dynamic risk stratification, and potentially treatment adaptation [47]. Hu et al. showed that pretreatment ctDNA levels demonstrated a significant correlation with tumor stage (*p* = 0.01) and showed that longitudinal changes in ctDNA can predict tumor progression with an area under the curve of 0.77. Notably, post-surgical ctDNA positivity identified molecular recurrence at a median of 90 days prior to radiographic detection and was independently associated with significantly shorter RFS (HR = 11.1, *p* = 0.006) and progression-free survival (PFS) (HR 12.6, *p* = 0.002) [48].

Further to its utility in prognostication, ctDNA offers a non-invasive method to capture real-time tumor molecular profiles [49]. ctDNA potentially overcomes the challenge of spatial and temporal heterogeneity that can limit tissue biopsies. Unlike single-site sampling, ctDNA reflects the full mutational landscape of the disease, allowing dynamic monitoring of clonal evolution and treatment resistance [50]. Evidence to date indicates that ctDNA is likely to be a powerful tool for precision oncology, facilitating not only risk stratification and early detection of recurrence for patients undergoing surveillance but also the real-time adaptation of therapy by longitudinal tracking of ctDNA. As such, it is likely that ctDNA may represent a potential component of future surveillance protocols in EGA. Emerging techniques such as the evaluation of methylation and fragmentomics and integrated artificial intelligence and machine learning bioinformatic analysis may further enhance the predictive accuracy of available technologies.

However, several barriers currently limit the routine use of ctDNA for post-treatment surveillance in EGA, including assay heterogeneity, variable tumor shedding and sensitivity, uncertainty regarding optimal sampling intervals, and differences between tumor-informed and tumor-agnostic approaches. When considering incorporation into surveillance programs, we must consider that false-negative results may provide inappropriate reassurance, while the optimal management of ctDNA positivity in the absence of radiologically detectable disease remains undefined, representing a significant clinical challenge in the absence of trial data to inform ctDNA actionability. Importantly, although ctDNA positivity is prognostic for recurrence, there is currently no evidence that ctDNA-guided imaging or therapy initiation improves overall survival in EGA, while data from ENSURE highlight the potential risks associated with intensive post-treatment monitoring, with greater disease-related anxiety and fear of cancer recurrence in this group [46,51]. Consequently, ctDNA should currently be considered an investigational surveillance strategy requiring prospective demonstration not just of prognostic value but also of clinical actionability, with cost and accessibility representing additional barriers to routine implementation.

### 7.2. CTCs in EGA

Another form of liquid biopsy with potential in surveillance of EGA is circulating tumor cells (CTCs), which are living tumor cells shed from the tumor found in the circulation and thought to be mechanistically central to haematogenous metastases [52]. Data indicate that CTC monitoring may have utility as a prognostic biomarker during post-treatment surveillance, although it is anticipated that sensitivity may be reduced as compared with ctDNA monitoring. Secondary analysis of the QUINTETT trial showed that detection or increase of CTCs in peripheral blood during follow-up in patients with non-metastatic esophageal cancer undergoing tri-modality therapy was associated with significantly worse DFS and OS [53].

### 7.3. Liquid Biopsy and Tumor Heterogeneity

One of the most exciting uses of liquid biopsy techniques such as ctDNA and CTCs is their potential to overcome the issue of temporal and spatial heterogeneity in EGA. The limited efficacy of molecularly targeted treatment in EGA may be partly explained by the frequent discordance observed between primary and metastatic tumors. Pectasides et al. showed that, in discordant primary and metastatic lesions, there was an 87.5% concordance for targetable alterations in metastatic tissue and cfDNA, suggesting a clear role for cfDNA profiling to enhance selection of targeted therapy [48,54]. In the future, liquid biopsy techniques may be incorporated into surveillance strategies. Other emerging technologies such as microRNAs are in evaluation and may represent new opportunities for post-treatment surveillance in EGA [55].

## 8. Rising Incidence of EGA in Younger Patients: Implications for Long-Term Surveillance

Epidemiological trends show a rising incidence of EGA in younger adults (18–49 years), who often present with advanced disease yet retain better performance status and fewer comorbidities than older patients [56,57]. This shift has important implications for post-treatment surveillance, as younger patients are more likely to be candidates for tumor-directed therapy in case of recurrence. This population also faces unique challenges in relation to cancer surveillance, particularly in terms of fear of recurrence, psychosocial needs, and HRQL. Aggressive tumor biology is more common in this group, including poorly differentiated, diffuse-type, or signet-ring histology [57]. However, there is currently no evidence that younger age alone justifies more intensive post-treatment surveillance or improves outcomes from such an approach.

## 9. Surveillance as a Gateway to Survivorship

Optimized surveillance can enable early detection of recurrence and timely access to targeted therapies, potentially prolonging survival in patients with EGA. Equally important, however, is the preservation of quality of life throughout cancer survivorship. Structured frameworks are therefore needed that monitor not only disease recurrence but also patients’ overall well-being, supporting a holistic survivorship model.

Patients’ voices must be central to the design of future surveillance strategies. Interestingly, a Dutch ‘thought experiment’ study previously showed that patients estimated they would prefer to have the routine imaging performed as compared with no imaging, even if such practices would not yield a survival benefit [58]. Conversely, the European multicenter observational ENSURE study found that although intensive surveillance was associated with higher levels of health-related anxiety, there was no difference in overall HRQL, suggesting that intensive surveillance itself may be associated with increased fear of cancer recurrence [31]. Beyond oncological surveillance itself, a comprehensive multidisciplinary approach is likely to benefit patients in EGA survivorship more broadly, with many units now developing survivorship services with embedded clinical nutrition support and physiotherapy-led rehabilitation [53].

## 10. The Role of Artificial Intelligence in Surveillance

Artificial intelligence (AI) has potential applications in post-treatment surveillance, particularly for recurrence-risk prediction. Machine learning and deep-learning models using clinicopathological and imaging data have demonstrated promising performance in predicting postoperative recurrence in gastric and esophageal cancer [59,60,61]. Radiomics and machine learning image analysis are increasingly utilized on other cancer types, with the incorporation of AI-supported imaging pathways already impacting practice in other cancers through well-designed clinical trials, particularly in breast cancer [62]. However, evidence sufficient to support clinical implementation in EGA is currently lacking, and large-scale trials are needed to determine the utility of AI to inform clinical decision making in EGA.

## 11. Future Research

Despite growing enthusiasm for intensive surveillance in EGA, current practices remain heterogenous and largely empiric, guided more by tradition and institutional preference than robust evidence. Randomized studies comparing different surveillance strategies, including symptom-based follow-up versus structured, multimodal surveillance, are urgently needed, and the results of the SARONG-I and -II randomized controlled trials are eagerly awaited [44]. With the evolution of precision oncology, future strategies must incorporate non-invasive tools for monitoring the molecular landscape of tumors, and liquid biopsy represents a key opportunity in this regard. Future surveillance strategies must also address cost-effectiveness within already strained public health systems while explicitly incorporating health equity considerations and anticipating global implementation barriers associated with emerging technologies.

## 12. Conclusions

There remains a lack of consensus on the optimal surveillance strategy for EGA. Ongoing high-quality prospective clinical trials will inform future clinical guidelines, which must also reflect the advancing precision oncology landscape and prioritize biologically informed approaches. Emerging technologies, including AI-driven risk modelling and liquid biopsy, may contribute to next-generation frameworks. Future research should evaluate these strategies within rigorously designed randomized controlled trials to establish clinical utility and cost-effectiveness.

## Figures and Tables

**Figure 1:**
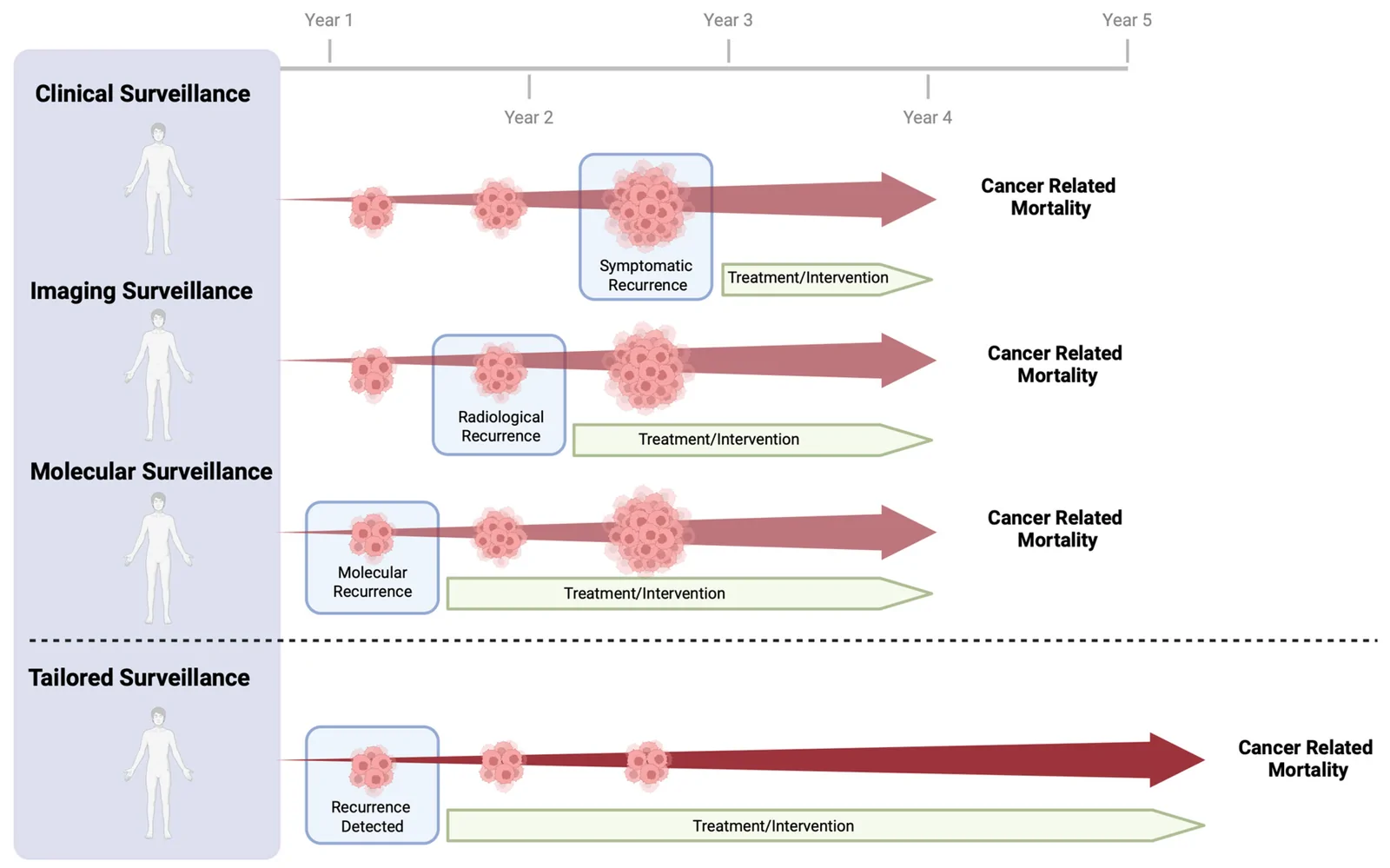
Lead Time Bias. This diagram illustrates how earlier detection of recurrence through surveillance (clinical, radiological, or molecular) can artificially extend disease-free survival (DFS) without improving overall survival (OS), a phenomenon known as lead time bias. The final panel contrasts this with a scenario showing a true survival benefit, where earlier intervention genuinely prolongs life expectancy.

**Figure 2:**
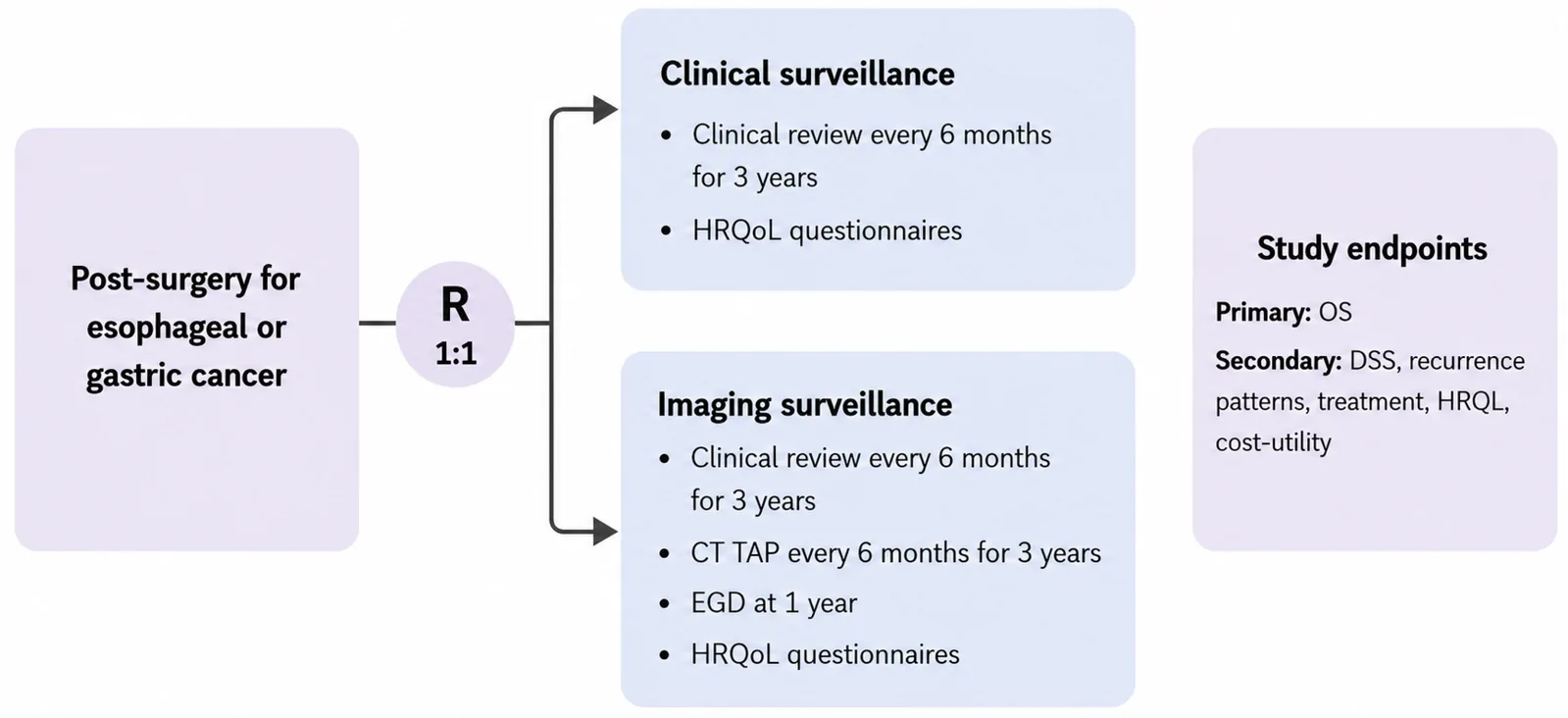
SARONG I and II Study Design. Patients undergoing curative-intent surgery for esophageal cancer are randomized (1:1) to either clinical surveillance or imaging surveillance. The clinical surveillance arm comprises clinical review every 6 months for 3 years with concurrent health-related quality of life (HRQoL) questionnaires. The imaging surveillance arm comprises clinical review every 6 months for 3 years, CT of the thorax, abdomen, and pelvis (CT TAP) every 6 months for 3 years, esophagogastroduodenoscopy (EGD) at 1 year, and concurrent HRQoL questionnaires. The primary endpoint is overall survival (OS). Secondary endpoints include disease-specific survival (DSS), recurrence patterns, treatment following recurrence, health-related quality of life (HRQoL), and cost–utility.

**Figure 3:**
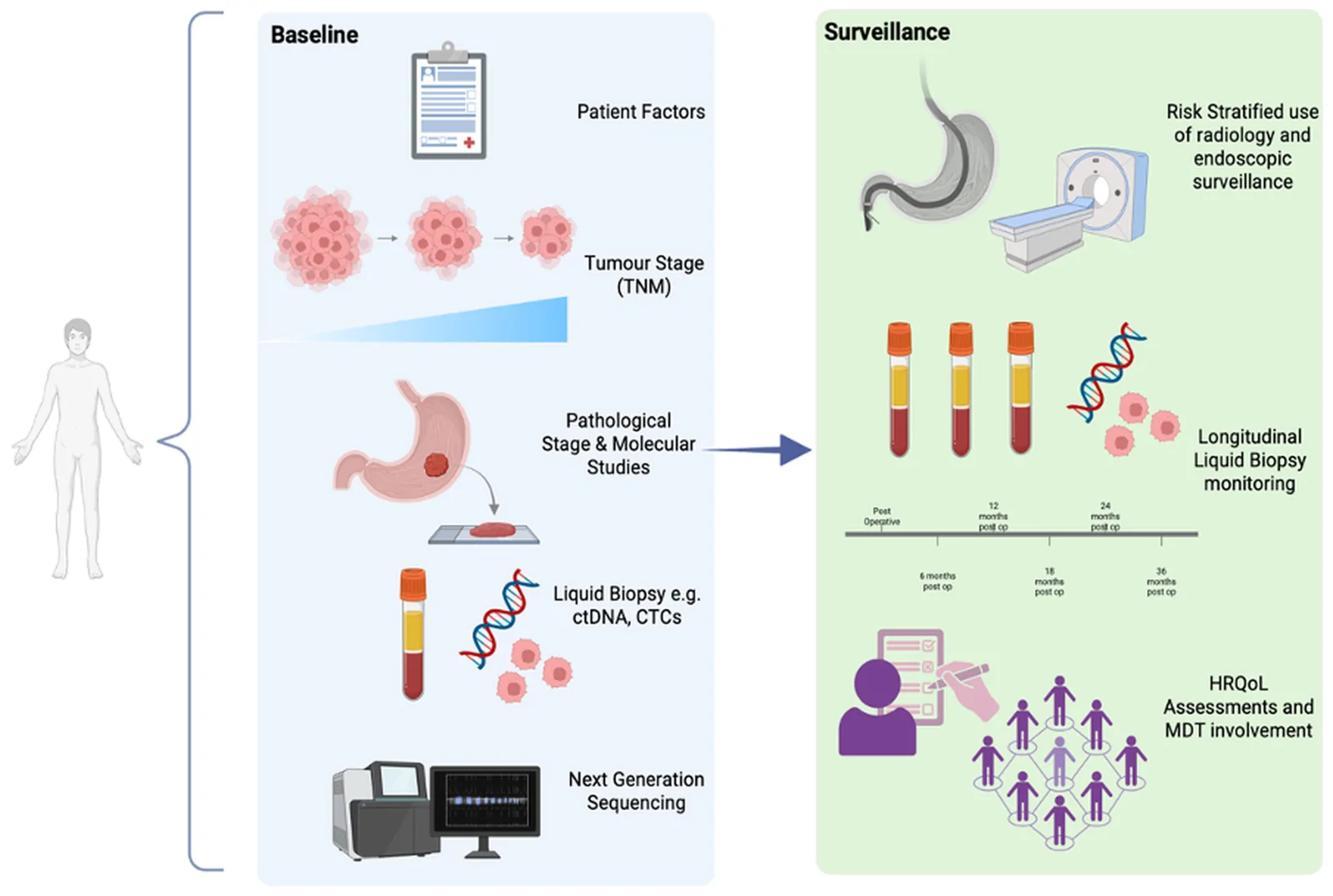
Future Surveillance Strategies. A comprehensive framework integrating baseline assessment and longitudinal monitoring for patients with EGA treated with curative intent. Initial evaluation includes detailed history, physical examination, work-up, staging investigations, surgery with pathological assessment, and baseline molecular profiling via liquid biopsy and tumor specimen for next-generation sequencing analysis. Post-treatment surveillance combines the risk-stratified use of endoscopic evaluation, CT imaging with longitudinal liquid biopsy for early detection of recurrence, health-related quality of life assessments, and ongoing multidisciplinary team input to guide patient care through survivorship and beyond CTC.

## Data Availability

No new data were created or analyzed in this study. Data sharing is not applicable to this article.

## Abbreviations

The following abbreviations are used in this manuscript:

AI	Artificial intelligence
CAD	Computer-aided diagnosis
CAPOX	Capecitabine and oxaliplatin
CEA	Carcinoembryonic antigen
cfDNA	Cell-free DNA
CI	Confidence interval
CLDN18.2	Claudin 18.2
CPS	Combined Positive Score
CT	Computed tomography
CTCs	Circulating tumor cells
ctDNA	Circulating tumor DNA
ddPCR	Digital droplet polymerase chain reaction
DFS	Disease-free survival
DNA	Deoxyribonucleic acid
DSS	Disease-specific survival
EAC	Esophageal adenocarcinoma
EGA	Esophagogastric adenocarcinoma
EGD	Esophagogastroduodenoscopy
EMA	European Medicines Agency
ESMO	European Society for Medical Oncology
EUS	Endoscopic ultrasound
FDA	Food and Drug Administration
FGFR	Fibroblast growth factor receptor
FGFR2	Fibroblast growth factor receptor 2
FGFR2b	Fibroblast growth factor receptor 2b
FLOT	Fluorouracil, leucovorin, oxaliplatin and docetaxel
FOLFOX	Fluorouracil, leucovorin and oxaliplatin
GEJ	Gastroesophageal junction
HER2	Human epidermal growth factor receptor 2
HR	Hazard ratio
HRQoL	Health-related quality of life
ICIs	Immune checkpoint inhibitors
MDT	Multidisciplinary team
ML	Machine learning
MMR	Mismatch repair
MRD	Minimal residual disease
MSI	Microsatellite instability
MSI-H	Microsatellite instability-high
MSI-L	Microsatellite instability-low
MSS	Microsatellite stable
NCCN	National Comprehensive Cancer Network
NGS	Next-generation sequencing
NICE	National Institute for Health and Care Excellence
OMD	Oligometastatic disease
OS	Overall survival
PD-1	Programmed death-1
PD-L1	Programmed death-ligand 1
PET	Positron emission tomography
PET-CT	Positron emission tomography–computed tomography
PFS	Progression-free survival
RFS	Recurrence-free survival
SCC	Squamous cell carcinoma
TAP	Tumor Area Positivity
TCGA	The Cancer Genome Atlas
TPS	Tumor Proportion Score
